# Ultrasound Common Carotid Artery Segmentation Based on Active Shape Model

**DOI:** 10.1155/2013/345968

**Published:** 2013-03-06

**Authors:** Xin Yang, Jiaoying Jin, Mengling Xu, Huihui Wu, Wanji He, Ming Yuchi, Mingyue Ding

**Affiliations:** ^1^State Key Laboratory for Multispectral Information Processing Technologies, Institute for Pattern Recognition and Artificial Intelligence (IPRAI), Huazhong University of Science and Technology (HUST), Wuhan, Hubei 430074, China; ^2^Department of Biomedical Engineering, College of Life Science and Technology, Image Processing and Intelligence Control Key Laboratory of Education of Ministry of China, Huazhong University of Science and Technology (HUST), Wuhan, Hubei 430074, China; ^3^Biomedical Instrument Institute, Med-X Research Institute, Shanghai Jiaotong University, Shanghai 200030, China

## Abstract

Carotid atherosclerosis is a major reason of stroke, a leading cause of death and disability. In this paper, a segmentation method based on Active Shape Model (ASM) is developed and evaluated to outline common carotid artery (CCA) for carotid atherosclerosis computer-aided evaluation and diagnosis. The proposed method is used to segment both media-adventitia-boundary (MAB) and lumen-intima-boundary (LIB) on transverse views slices from three-dimensional ultrasound (3D US) images. The data set consists of sixty-eight, 17 × 2 × 2, 3D US volume data acquired from the left and right carotid arteries of seventeen patients (eight treated with 80 mg atorvastatin and nine with placebo), who had carotid stenosis of 60% or more, at baseline and after three months of treatment. Manually outlined boundaries by expert are adopted as the ground truth for evaluation. For the MAB and LIB segmentations, respectively, the algorithm yielded Dice Similarity Coefficient (DSC) of 94.4% ± 3.2% and 92.8% ± 3.3%, mean absolute distances (MAD) of 0.26 ± 0.18 mm and 0.33 ± 0.21 mm, and maximum absolute distances (MAXD) of 0.75 ± 0.46 mm and 0.84 ± 0.39 mm. It took 4.3 ± 0.5 mins to segment single 3D US images, while it took 11.7 ± 1.2 mins for manual segmentation. The method would promote the translation of carotid 3D US to clinical care for the monitoring of the atherosclerotic disease progression and regression.

## 1. Introduction

Cardiovascular disease (CVD) is the leading cause of death globally based on the World Health Organization (WHO, 2009). The main precursors of CVD are smoking, obesity, hypertension, and a disturbed serum lipid profile [1]. The therapy evaluation and clinical data analysis are important to the cerebrovascular and cardiovascular pathologies diagnosis. Because thrombosis generation and subsequent cerebral emboli have a high risk leading to death, carotid atherosclerosis is becoming a significant issue for researches recently [2]. Measurement accuracy and geometric understanding of common carotid artery (CCA) play an important role in carotid atherosclerosis assessment and management [3], which requires precise segmentation.

Ultrasound (US) has been employed as a routine examination for inexpensive and noninvasive clinical diagnosis of atherosclerosis (the hardening of the arteries) [4, 5]. Furthermore, the three-dimensional ultrasound (3D US) [6] imaging can be used to quantitatively monitor carotid plaque progression or regression without ionizing radiation [7–10].

**Figure 1:**
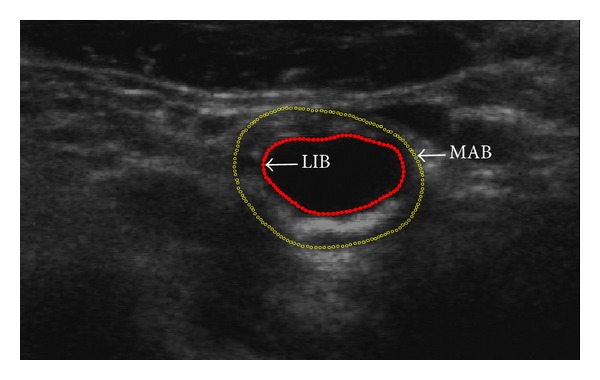
US image of a carotid artery with the expert-drawn contours delineating the LIB (red solid contour) and MAB (yellow hollow point contour).

Among various US image segmentation methods for carotid atherosclerosis quantification [11–15], intima-media thickness (IMT) measurement is proved to work efficiently on longitudinal views of two-dimensional (2D) US images [16]. The media-adventitia boundary (MAB) and lumen-intima boundary (LIB) can be extracted simultaneously. This work focuses on the MAB and LIB segmentation on the transverse views US images and is expected to reduce diagnosticians' manual workload (Figure 1). The potential applications mainly include patient management, genetic research, and therapy evaluation [17].

Several automatic and semiautomatic methods for the segmentation of LIB and/or MAB on 2D transverse US images have been reported [18, 19]. Mao et al. [20] proposed a deformable contour model approach for carotid LIB semiautomated segmentation. One seed point was located on the image by the user firstly. Then, the local gradient difference was obtained with local grey level ratio between the exterior and interior of the deformable contour. The method was validated by a group of seven B-mode US images. Abolmaesumi et al. [21] presented a method to fulfill real-time extraction of carotid LIB on 2D US image sequences. They modified the star algorithm by using a temporal Kalman filter to track the center of LIB and adopting a spatial Kalman filter to extract LIB contour. Digitized US video images were used for validation. Zahalka and Fenster [22] introduced a carotid LIB segmentation method on 3D US images with geometrically deformable model (GDM). Li et al. [23] also developed a GDM with automatic merge function to segment carotid contours, but only tested their method on simulated 3D US images. A scheme for detecting the normal regions in carotid artery US images was proposed by Balasundaram and Banu [24]; however, no process was taken to remove the noise in the images. Lou and Ding [25] used particle motion mechanics to segment object boundaries. The method was sensitive to noise. Fast Marching Method (FMM) [26] originally for intravascular ultrasound (IVUS) image segmentation was also adopted for vascular US image segmentation. Common carotid artery (CCA) boundary identification pipeline, a simple and effective method, was proposed according to mathematical morphology [27], but it was only tested for limited lumen boundaries segmentation. The latest segmentation method for carotid MAB and LIB in transverse US images was proposed by Ukwatta et al. [28]. They adopted an active contour based on level set method. A combination of image information from energy, geodesic energy, and anchor constraint energy was used to drive the deformable contour to the desired one. However, the global optimum of the searching parameters cannot be guaranteed. Other studies by application of regional growing [29], diffusion-based filters [30], edge detection combined with morphology methods [31], and Hough transforms [32], were also reported.

Our purpose is therefore to develop and validate a new segmentation approach, which would be used to delineate the lumen-intima boundary (LIB) and media-adventitia boundary (MAB) of the common carotid artery (CCA) from 3D US images. The key innovation of this work is applying the Active Shape Model (ASM) segmentation to two separate time points, which used baseline data for training and follow-up data for segmentation. And the technology enables the accurate, inexpensive, and noninvasive method for progression and regression monitoring of atherosclerosis and drug therapy evaluation.

The following of this paper is organized as follows. In Section 2, the proposed method is explained in details. The results are shown in Section 3. Sections 4 and 5 will contain the discussion and conclusion.

## 2. Methods

**Figure 2:**
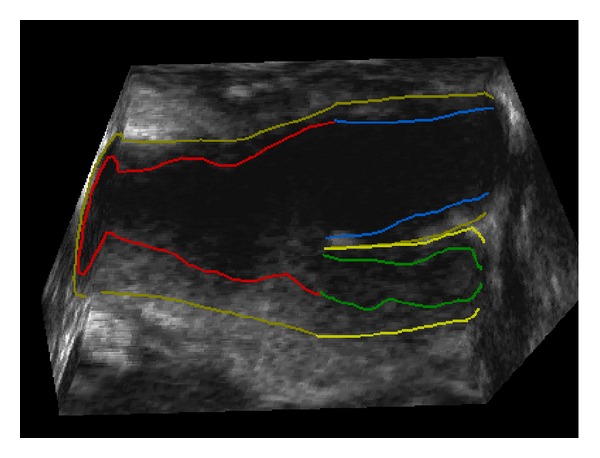
Three-dimensional ultrasound carotid artery longitudinal view in clinical trials [40]. Both baseline and follow-up 3D images, constructed from the set of 2D frames, were examined simultaneously to visually match the bifurcation (BF) points in both images by an operator blinded to time point and treatment. Each 3D US image was manually segmented starting from the bifurcation point extending into around 10–15 mm of common carotid artery (CCA) and about 10 mm into internal carotid artery (ICA) at 1 mm interval perpendicular to the artery axis; refer to Figure 3. This study was only carried out on the CCA, since the focus was on stroke risk.

The proposed algorithm is validated by comparing the LIB and MAB segmentation results with the manual ones from the expert. The typical US images used in this paper are shown in Figures 1 and 2. Figure 1 shows a transverse view of a CCA with manually annotated MAB and LIB boundaries superimposed.  Figure 2 shows the surface of the manual segmented inner and outer walls, including CCA, internal carotid artery (ICA), external carotid artery (ECA), and carotid sinus (bifurcation (BF)).

The atheromatous plaque has been well described in terms of its progression, and the clinical characterization of the atherosclerotic lesion has also been well documented [33]. Furthermore, it is true that plaques (a) extend into the internal carotid artery and (b) that rupture of these plaques will lead to stroke which has been well documented. However, the vulnerable plaque in terms of its concepts is still a novel area [34]. “Vulnerable plaque” is a term that has been derived from a subgroup known as stenotic plaques. They are prone to both rupture and erosion, sometimes causing acute coronary syndromes and sudden cardiac death. Rupture prone plaques have been shown in postmortem evaluation to have specific characteristics [35]. Depending on the severity level of the plaque, dietary change, drug treatment, or eventually surgical treatment such as carotid endarterectomy (CEA) may be introduced to prevent major heart attacks or strokes.

As will be described in the following sections, the assessment of plaque vulnerability and risk of potential rupture is very difficult noninvasively [36, 37]. And the three-dimensional (3D) US vessel wall volume (VWV) measurement is a 3D measurement of the vessel wall thickness plus plaque within the carotid arteries. 3D US VWV measurements are sensitive to changes in both intima-media thickness and plaque and thus provide alternative and complementary information to IMT [38, 39]. The MAB and LIB segmentation on cross-section is a vital step for both qualitative and quantitative evaluation. In most cases, the CCA can be used to reflect and evaluate the carotid atherosclerosis severity much more comprehensively and accurately than ICA and ECA; this may be due to the significant amount of plaque present proximal to the BF of the carotid artery. Therefore, the proposed segmentation method was only carried out on the CCA, since the focus was on the essential part of stroke risk.

In this study, we only segmented a portion of the common carotid artery (CCA). However, in the future, we will investigate the segmentation of the internal and external carotid arteries (ICA, ECA) as well. The proposed algorithm segments each transverse slice independently and is a first step toward reducing the operator interaction for carotid segmentation. As for a future work, we will investigate the use of both slice-by-slice propagation and direct 3D segmentation to reduce the operator interaction further by utilizing the image information along the out of plane direction as well.

### 2.1. Image Acquisition

The mechanical 3D US system utilized in this study was described previously in [40]. The images were acquired by driving a linear ultrasound transducer (L12-5, Philips, Bothell, WA, USA, 8.5 MHz central frequency) with a motorized linear device along the neck of the subject at a uniform speed of 3 mm/s for about 4 cm without cardiac gating [6].

The 2D ultrasound frames were captured by the US machine (ATL HDI 5000, Philips, Bothell, WA, USA) and reconstructed to 3D images with 3D Quantify (a multiplanar visualization software) [41]. The voxel size was approximately 0.1 × 0.1 × 0.15 mm^3^.

**Figure 3:**
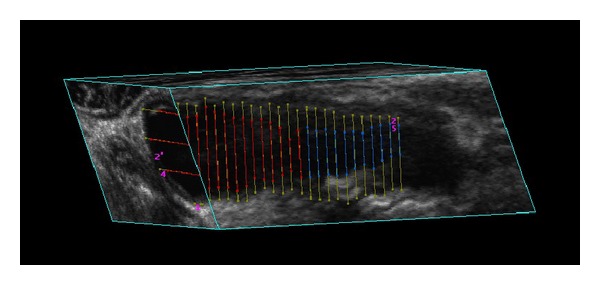
Sagittal cross-section of a common carotid artery (CCA) in 3D US image. The contours on the image show the manual delineations done by the physician. The inner boundary is lumen-intima boundary (LIB), and the outer boundary is the media-adventitia boundary (MAB). The segmentations were performed on parallel images with interslice distance (ISD) 1 mm.

The 3D Quantify generates 2D images of the artery by slicing through the 3D image orthogonally to the medial axis, in the inferior direction from the bifurcation (BF), with an interslice distance (ISD) of 1 mm (Figure 3). 

### 2.2. Study Subjects

Seventeen patients with carotid stenosis over 60% were enrolled in this study [6]. The presence of stenosis was verified using carotid Doppler US flow velocities. 8 subjects, 4 males and 4 females with mean age ± SD (65 ± 6.6  years), were supplied with 80 mg atorvastatin daily for 3 months. The remaining 9 subjects, 4 males and 5 females with mean age ± SD (68 ± 8.4  years), were assigned to the placebo. Baseline and follow-up (3 months later) 3D US images were acquired for each subject, for both left and right carotid arteries. All subjects, in this study, were recruited from the Premature Atherosclerosis Clinic and the Stroke Prevention Clinic at University Hospital (London Health Sciences Center, London, Canada) and the Stroke Prevention and Atherosclerosis Research Center (Robarts Research Institute, London, ON, Canada). 

A written informed consent of the study protocol approved by the University of Western Ontario Standing Board of Human Research Ethics was provided to all subjects.

### 2.3. Manual Segmentation

Manual segmentation of CCA boundaries is labor intensive and time consuming [42]. There are several studies on semiautomated segmentation methods for delineating carotid walls with 2D US images [21].

The manual segmentation method used in our work was proposed by Egger et al. [18]. Prior to contouring, the expert first located the BF and defined an approximate medial axis of the carotid artery by choosing two end points of the axis. The multiplanar 3D viewing software then presented 2D images of the artery by slicing through the 3D image orthogonally to the medial axis, in the inferior direction from the BF, with an ISD of 1 mm. The expert then performed contouring of arteries on each of these images. Figure 1 showed a transverse view of a common carotid artery with manually annotated boundaries overlaid. An expert outlined the vessel boundaries for five times within one-day intervals. The image sequences were randomized, and the operators were blinded to the image order during each repetition to reduce memory bias [28].

### 2.4. Preprocessing

Several preprocessing steps were applied prior to LIB and MAB segmentation. Firstly, contrast limited adaptive histogram equalization (CLAHE) [43] was applied to enhance the local contrast of the US image. CLAHE partitioned the images into contextual regions and applied histogram equalization by fitting a Rayleigh distribution to each region [44]. Next, Speckle Reducing Anisotropic Diffusion Method (SRAD) was used for US speckle noise reduction [45]. The SRAD was used to enhance the edges by inhibiting diffusion across edges and allowing diffusion on either side of the edges.

### 2.5. Active Shape Model (ASM)

Active Shape Model (ASM) is one of the statistical shape models (SSMs) developed by Cootes et al. in 1995 [46]. The shape of an object is usually represented by a set of *n* points in ASM. By analysing the variations in shape, a statistical model is built which can mimic the variation [47]. The ASM algorithm seeks to match a set of model points to an image, constrained by the statistical model of shape which learns the valid ranges of shape variation from the training set of labelled images [48]. The general working steps of ASM are as follows: (a) look in the image around each point for a better position for that point (to locate a better position for each point one can look for strong edges, or an expected match to a statistical model at the point); (b) compute the changes in the pose and shape parameters based on (a); (c) update the model parameters to improve the match between a shape model and image instance to ensure the model only deforms into shapes consistent with the training set.

**Figure 4:**
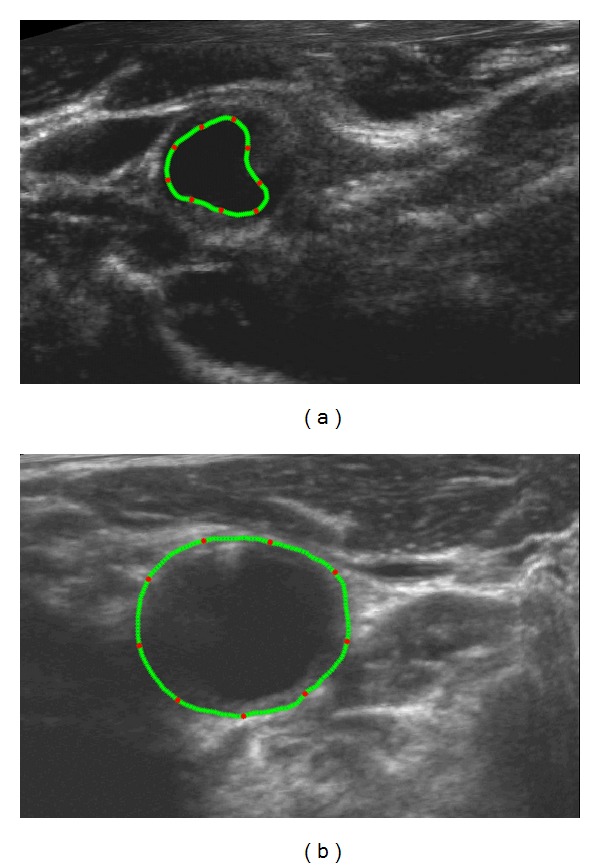
Nine equally spaced landmark points (red points) along the manual contour (green points) were averagely picked for ASM training ((a): LIB; (b): MAB; refer to Figure 1). Three hundred and forty images were labelled by senior physicians. Some of the points may locate on weak edges.

The technique is widely used to analyse images of faces, mechanical assemblies, and medical images in 2D applications. Given a rough starting approximation (*X* ^0^), the ASM matches the CCA model points to a new image using an iterative technique. An ASM is defined by (1):

(1) $$\begin{array}{c} X=\overset{-}{X}+P\cdot b, \end{array}$$

where $\overset{-}{X}$ represents the mean shape of the training set, *P* is a matrix of the first few principal components of the shape, created by using Principal Component Analysis (PCA), and *b* is shape parameters for the model, along with parameters defining the global pose (the position, orientation, and scale) [48], whose standard deviation from the mean shape ranges between −3 and +3. Therefore, *X* is defined by the variable *b*. Given a set of landmark points *X* ^*i*^ for iteration *i*, the goal is to find the landmark points $\dot{X^{i}}$ closest to the object border. The shape is then updated by (2):

(2) $$\begin{array}{c} b=P^{T}\cdot \left(\dot{X^{i}}-X^{i}\right), \end{array}$$

where each element of *b* can only be within ±3 standard deviations of the mean shape. The final ASM segmentation is denoted as *X* ^Final^. The training set of the ASM to determine $\overset{-}{X}$ and *P* is performed by manual delineation of the artery boundaries followed by manual alignment of 9 equally spaced landmark points (red points) along the contour (green points) on both LIB and MAB as shown in Figure 4. 

**Figure 5:**
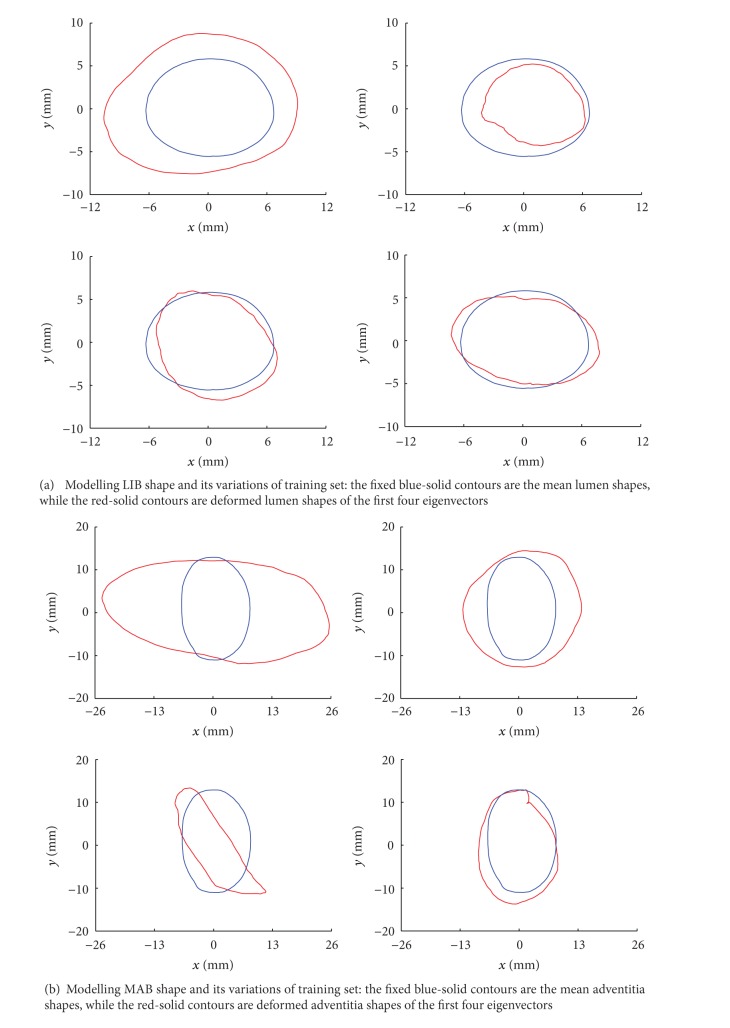
Mean shapes in each panel are the fixed (in blue); the first four eigenvectors-corresponded deformed shapes are diverse (in red), because of the variety shapes of the LIB (a) and MAB (b) of training set. The units of both *x*- and *y*-axes of every subpicture are (mm). The training results are generated from three hundred and forty 2D CCA baseline images data. The blue contour is the average shape of baseline images data (training set), while the red one is the deformed shapes. The average shape contour would be superposed on follow-up images data (test set) as the initialization contour for ASM segmentation (refer to movie I in Supplementary Material available online at http://dx.doi.org/10.1155/2013/345968).

**Figure 6:**
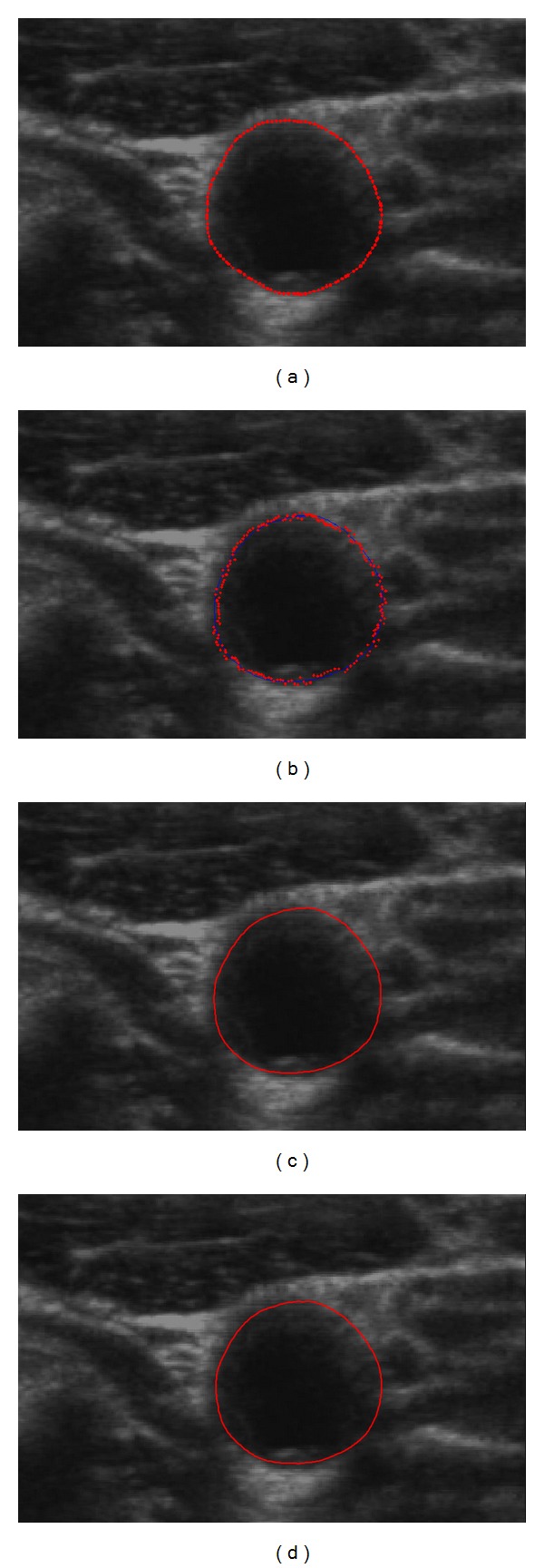
Adventitia results comparison after the three-month therapy: (a) original image with the manual segmentation result as a golden standard; (b) ASM segmentation process (refer to movie II in Supplementary Material available online at http://dx.doi.org/10.1155/2013/345968); (c) segmentation result of the proposed method; (d) segmentation result of the training set without the example data.

It should be noted that once the ASM is trained with the training set, it can be used for the new CCA image segmentations without significant manual intervention. Six hundred and eighty 2D CCA images in total, extracted from the 3D US data (10 two-dimensional images per each of 17 patients of two sides at 2 time points), have had their arterial walls manually segmented previously as the golden standard. Three hundred and forty (10 × 17 × 2) 2D CCA baseline images data and manual boundaries results were used for ASM learning as shown in Figure 5, while another three hundred and forty treatment images data were used for ASM segmentation and evaluation. And a demonstration of MAB segmentation progress is shown in Figure 6.

CVD morbidity and mortality rates are higher in atherosclerosis patients than in the general population [49], leading to a reduced lifespan, lower quality of life, and increased medical expenditures. Cross-sectional studies have shown that underlying these higher CVD rates is a greater burden of atherosclerosis in both the coronary [50] and carotid [51] vasculature of patients. Therefore, imaging technique needs to be considered to monitor substantial plaque progression or regression of atherosclerosis [52], even though the progression or regression of the disease may be significant or not between the two time points [6]. 

The main findings in the study [53] is that dietary interventions can induce a significant regression of carotid atherosclerosis, which could be detectable by B-mode and 3-dimensional ultrasound (3D US). What is more, based on the research of [40], the change is also significant during three-month statin treatment (atorvastatin) [54].

Since carotid anatomy varies considerably within individuals between left and right carotid arteries even in two different time points, which affects the development of plaque, and the low correlation coefficient between the left and right sides, each carotid artery can be considered an independent object [55]. Thus, the data sets used in this study could be considered as different objects, which were lowly correlated ones with statistical significance significant change.

Also, the authors would use completely different slices from various scans of diverse patients in future work as totally uncorrelated individual data to thoroughly separate the test and training data sets. And a preliminary test results showed that there was no obvious subjective differences, from which the training set is within (Figure 6(c)) and without (Figure 6(d)) the example baseline data.

Furthermore, external or internal carotid arteries (ECA, ICA) stenosis is less frequent and clinically less important than CCA stenosis, and the segmented contours of CCA could be used for drug treatment evaluation between baseline time and follow-up time.

### 2.6. Evaluation Metrics

The Dice Similarity Coefficient (DSC) was used as a region-based measure to compare segmentation results on slice-by-slice basis. The DSC quantifies the overlapping areas of two segmentation methods by the following equation (3):

(3) $$\begin{array}{c} \text{DSC}=2\frac{\left|R_{M}\cap R_{P}\right|}{\left|R_{M}\right|+\left|R_{P}\right|}, \end{array}$$

where *R*_*M*_ and *R*_*P*_ denote the region of the manual and proposed method boundaries, respectively.

The mean absolute distance (MAD) and maximum absolute distance (MAXD) were used as boundary distance-based metrics. The averages of MAD (see (4)) and MAXD (see (5)) were computed using all vessels in the testing images to obtain an overall estimate of boundary disagreement. And the computational time is also estimated:

(4) $$\begin{array}{c} \text{MA}{\text{D}}_{M,T}=\frac{1}{K}\sum_{i=1}^{K}\left|d\left(m_{i},T\right)\right|, \end{array}$$

(5) $$\begin{array}{c} \text{MAX}{\text{D}}_{M,T}=\underset{i\in \left[1,K\right]}{\max}\left\{\left|d\left(m_{i},T\right)\right|\right\}, \end{array}$$

where *d*(*m*_*i*_, *T*) is the distance between the vertex *m*_*i*_ of the manual drawn contour and its corresponding vertex on ASM contour *T*, and *K* is the number of vertices. 

## 3. Results

**Figure 7:**
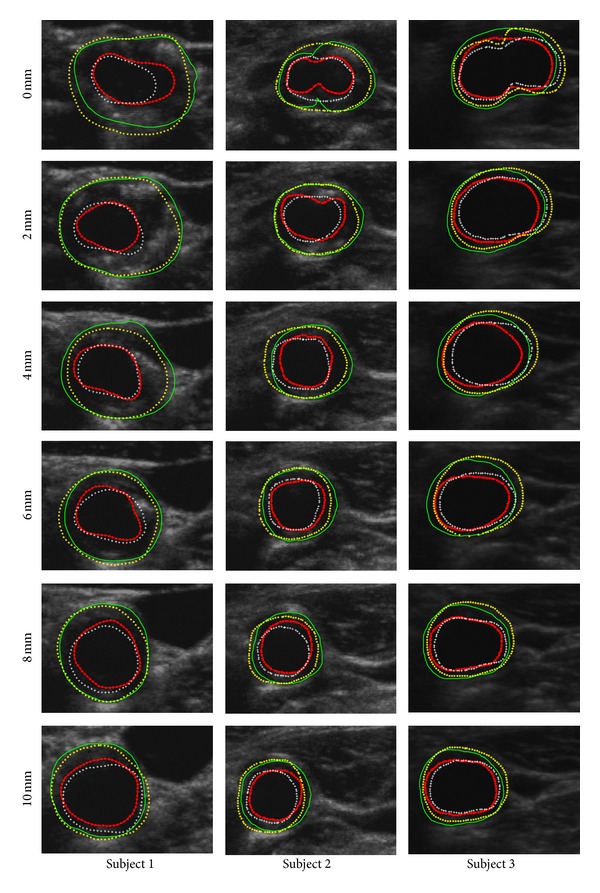
In order to subjectively and objectively evaluate the performance between the proposed approach and manually method, both inner and outer contours are synchronized overlapped together. Green-solid-line outer contour and red-dotted-line inner contour represent manual MAB and LIB, respectively; while yellow-starred outer contour and white-plus-signed inner contour represent algorithm-generated MAB and LIB, respectively. Each column represents the images of individual patient. For each row from the top to the bottom, there are images at different distance of 0, 2, 4, 6, 8 and 10 mm from the slice to the bifurcation (BF).

Figure 6 shows the MAB segmentation progress after follow-up treatment.  Figure 7 shows the segmentation results of 18 slices with the proposed approach and manually contours for 3 subjects with a moderate level of plaque.

### 3.1. Validation

The validation of our segmentation algorithm will require comparison with manual segmentation results. The accuracy, variability, and reproducibility of the proposed algorithm were evaluated by comparing with the physician-drawn contours. Three to five experts delineated the CCA boundaries on 340 2D slices. The method of Chalana and Kim [56] was used to compute the mean boundary from the repeated manual and algorithm-generated segmentations. The ordering of the images was randomized to reduce learning effects.

DSC, MAD, and MAXD were computed from 3D US images to obtain overall estimates of each metric for the image set.  Table 1 shows the overall evaluation results of the proposed algorithm for 340 transverse 2D US slices extracted from 17 subjects after treatment.

The proposed method yielded a DSC of 94.4% ± 3.2% and 92.8% ± 3.3% for the MAB and LIB, respectively. The method gave submillimeter error values for the MAD of 0.26 ± 0.18 mm and 0.33 ± 0.21 mm and MAXD of 0.75 ± 0.46 mm and 0.84 ± 0.39 mm for the MAB and LIB, respectively. Our approach took 4.3 ± 0.5 mins comparing to 11.7 ± 1.2 mins of operator processing time for manual segmentation to initialize/delineate a single 3D image [44].

## 4. Discussion

ASM performs exceptionally well when compared to other deformable models, especially when segmenting objects that do not have a clear, continuous boundary like the CCA. It can capture expert prior knowledge in the training examples annotation and compare resulting shapes easily simultaneously, since they have a strict point correspondence between landmark points. Therefore, ASM has been extensively explored and still under investigation as well in methodological aspects as in concrete applications.

The ASM algorithm is an iterative approach. It would be applied to the training set of CCA arteries to locate the other arteries. Assuming there is not a big difference between the frames, the shape for one frame can be used as the starting point for the search in the next, and it will require a few iterations to lock on. This approach is particularly useful for cases where the objects have a well-defined shape with a representative and available set of examples [57]. In medical image segmentation, because of the complexity of human anatomy and the volatility of the appearance, traditional approaches cannot obtain desired results. It requires a flexible framework which can combine the properties of the image itself with its prior knowledge.

In this paper, we introduced the ASM segmentation method to delineate the LIB and MAB boundaries of the CCA on transverse view sliced from 3D US images. The proposed method was evaluated by comparing the resulting boundaries and expert manually outlined boundaries which act as a surrogate for ground truth. The algorithm yielded a higher DSC for the MAB than for the LIB, and the algorithm gave similar MAD and MAXD errors for both vascular walls. It was obvious that the adventitia value is better than lumen, which implies that we got the better MAB segmentation result. The observations may result from (a) weak image edges, particularly on boundary segmentation that are parallel to the US beam direction and are not hard for ASM learning and segmentation; (b) different components between the two layers caused the different performance. Inside lumen is liquid blood, while the outside adventitia is complex connective tissue from the view of CCA physiology; (c) the initialized average contours from baseline training data have differences with the test data.

Most previous studies of the relationship between hemodynamic factors and plaque stability have used in vitro models. It is difficult to determine how accurate these models represent conditions in vivo.

By studying carotid bifurcation angiograms, Schulz and Rothwell [58] found the relationship between vessel anatomy and plaque stability in vivo. And they have shown that carotid anatomy varies considerably between individuals and can be very asymmetrical within individuals, which is not similar to faces or hands at all. Although it is possible that the variation in arterial anatomy might influence training and test results, there have been no previous studies of the association between arterial anatomy and ASM algorithm stability in either the coronary or carotid circulations of atherosclerosis.

Ideally, a study of the association between carotid anatomy and statistical model would require a large community-based cohort imaged with double-blinded randomly selected. Unfortunately, given the relatively low prevalence of moderate or severe carotid disease in the community (only dozens of cases) is not possible. And training and testing in this study may be performed within a round-robin (leave-one-case-out) protocol later.

The ASM approach has been demonstrated in 2D data. Our future work will extend it to 3D application. More 3D data needed to be collected. In addition, the definitions of surfaces and 3D topology are more complex than those required for 2D arteries boundaries. However, 3D models which represent shape deformation can be successfully used to locate structures in 3D data sets.

## 5. Conclusion

The main purpose of this work was to develop and evaluate a new segmentation algorithm for outlining both MAB and LIB of CCA on 2D transverse views sliced from 3D US images. From a quantitative evaluation of the results, we concluded that the proposed method could accurately segment the CCA and also the saved time on average was substantial.

We have used the point distribution model to represent an object as a set of labelled points, giving their mean positions and a small set of modes of variation. Applying limits to the parameters of the model enforces global shape constraints. The constraints ensure that the properties of the testing one are similar to those of the training set. Given a set of shape parameters, ASM can match the generated model to a new similar image rapidly.

Preliminary experimental results showed that the segmented areas could accurately define the locations of CCA contours. This method could save the physicians' time. Our work provides an easy-handle technique to simplify the job of labeling the contours in CCA manually. Therefore, it would be helpful to promote the translation of 3D carotid US to clinical care for the fast, safety, and economical monitoring of the atherosclerotic disease progression and regression during therapy.

In this method, the segmentations of the MAB and LIB can be used as a fundamental step in the analysis of carotid plaque composition for the early identification of vulnerable plaques and treatment evaluation to prevent a possible stroke [40]. The proposed approach has another merit, as clinical trials will be carried out temporal continuity on the same patients by serially imaging them. Thus, manual segmentation of the first time point followed by the authors, more automated method would save analysis time.

Future work would be focused on (a) ICA and ECA segmentation, (b) directly artery segmentation, and (c) thoroughly reducing the potential correlation between training and test sets for reasonable double-blinded test.

## Supplementary Material

Supplementary movie I: It includes both training and testing for ASM CCA segmentation. Mean shape (blue) is generated by the training dataset (green contours with red points). Final contour (red) of testing dataset is generated from the mean shape evaluation (blue contour with red points).
Supplementary movie II: It is an ASM MAB segmentation process demonstration. Locate the mean shape (blue) from training dataset on the artery from the test dataset, and potential contour (blue contour with red points) will automatically stop iteration at the final contour (red).

## Figures and Tables

**Table 1:** Overall performance results of the proposed algorithm. Validation results of segmentation for 340 transverse slices of both left and right sides from seventeen subjects (eight with 80 mg atorvastatin and nine with placebo, resp.,) after three-month treatment.

Metric	DC (%)	MAD (mm)	MAXD (mm)
Media-adventitia boundary (MAB)	94.4 ± 3.2	0.26 ± 0.18	0.75 ± 0.46
Lumen-intima boundary (LIB)	92.8 ± 3.3	0.33 ± 0.21	0.84 ± 0.39
